# MicroRNA-200a induces immunosuppression by promoting PTEN-mediated PD-L1 upregulation in osteosarcoma

**DOI:** 10.18632/aging.102679

**Published:** 2020-01-24

**Authors:** Zhuochao Liu, Junxiang Wen, Chuanlong Wu, Chuanzhen Hu, Jun Wang, Qiyuan Bao, Hongyi Wang, Jizhuang Wang, Qi Zhou, Li Wei, Yuhui Shen, Weibin Zhang

**Affiliations:** 1 Department of Orthopaedics, Ruijin Hospital, Shanghai Jiaotong University School of Medicine, Shanghai 200025, China; 2 Shanghai Institute of Orthopedics and Traumatology, Ruijin Hospital, Shanghai Jiaotong University School of Medicine, Shanghai 200025, China; 3 Department of Orthopaedics, Shanghai Tenth People’s Hospital, Tongji University, Shanghai 200072, China

**Keywords:** microRNA-200a, PTEN, PD-L1, CD8+ T cells, immunosuppression

## Abstract

In this study, we identified microRNAs that regulate the expression of programmed death-ligand 1(PD-L1) in osteosarcoma and investigated their role in PD-L1-targeted immunotherapy. MicroRNA sequencing analysis showed that the expression of PD-L1 is regulated by microRNA-200a in U2OS, 143B, and K7 osteosarcoma cells. MicroRNA-200a overexpression induced the upregulation of PD-L1 in the osteosarcoma cells. CD8^+^ T cells co-cultured with microRNA-200a-overexpressing osteosarcoma cells showed reduced survival, proliferation, and secretion of granzyme B and perforin. The same phenomenon was also observed in the K7-derived syngeneic mouse model, as microRNA-200a promoted tumor growth by increasing the percentage of Foxp3^+^ regulatory T lymphocytes while reducing the proportions of CD4^+^, CD8^+^, and IFN-γ^+^ cytotoxic T lymphocytes. But microRNA-200a overexpression group was also more responsive to PD-L1-targeted immunotherapy than the controls. In addition, the tumor tissues from 32 osteosarcoma patients showed that high expression of microRNA-200a and PD-L1 was associated with poor tumor necrosis rate after chemotherapy. Moreover, we confirmed that tensin homolog deleted on chromosome ten (PTEN) could act as the target gene for microRNA-200a during the upregulation of PD-L1. Thus, our findings provide important and novel insight into a regulatory axis involving microRNA-200a/PTEN/ PD-L1 axis, which determines osteosarcoma growth and the efficacy of PD-L1-targeted immunotherapy.

## INTRODUCTION

Osteosarcoma is the most common primary malignancy of bones in children and adolescents [1]. In the 1970s, the application of chemotherapy substantially improved the 5-year survival rate of osteosarcoma patients [2]. However, due to immunosuppression induced by chemotherapeutic agents such as cisplatin and doxorubicin, as well as the lack of new chemotherapeutic drugs owing to the heterogeneity of osteosarcoma, there has been no significant improvement in the 5-year survival rate since then. Hence, newer therapeutic strategies are needed to improve survival rates of osteosarcoma patients.

Programmed death-ligand 1 (PD-L1) plays an important role in chemotherapy-related immunosuppression. Our previous study suggested that doxorubicin treatment would cause immunosuppression by inducing PD-L1 expression [3]. The high expression of PD-L1 predicts a poorer 5-year event-free-survival of osteosarcoma patients [4] and is correlated with early metastasis [5]. And the disruption of PD-L1 by CRISPR/Cas9 *in vitro* could enhance the sensitivity of osteosarcoma cells to conventional chemotherapy [6]. Although PD-L1 is a promising target for immunotherapy in osteosarcoma patients [7], PD-L1-targeted immunotherapy is not effective despite the high PD-L1 positive rate in the tumor tissues [4, 8]. This suggests that PD-L1 positivity alone is not a reliable indicator of the efficacy of PD-L1-targeted immunotherapy. Hence, factors that involved in the regulation of PD-L1 expression in osteosarcoma need to be identified so as to improve the efficacy of immunotherapy against PD-L1.

The expression of PD-L1 is influenced by several factors, including chemotherapy [3], cancer-promoting signaling pathways [9, 10] and microRNAs [11]. Recent studies suggest that microRNAs (miRNAs) are closely related to the modulation of PD-L1 expression. In non-small cell lung cancer (NSCLC), miR-140 and miR-142 suppress the expression of PD-L1 by directly targeting its 3’UTR [12, 13]. Whereas in gastric cancer, miR-940 enhances the expression of PD-L1 by targeting its negative regulator, Cbl-b [14]. Moreover, miRNAs exert a considerable influence on tumorigenesis by affecting the tumor phenotype [15] and anti-tumor immunity [16]. In this study, we investigated the miRNAs that regulate the expression of PD-L1 in osteosarcoma and their relevance to the efficacy of PD-L1-targeted immunotherapy.

## RESULTS

### miRNA-200a promotes doxorubicin-induced PD-L1 upregulation in osteosarcoma cells

**Figure 1:**
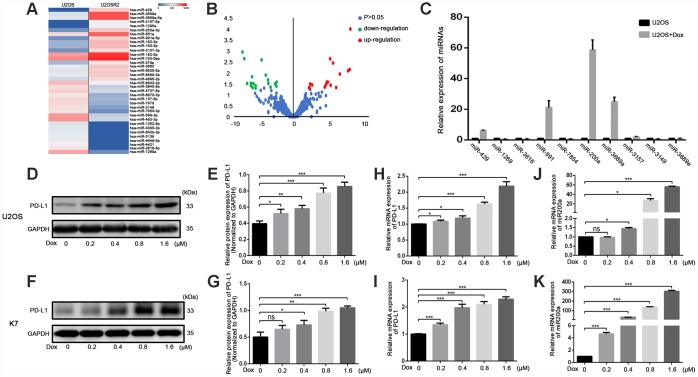
**Doxorubicin-induced the simultaneous upregulation of PD-L1 and miR-200a in osteosarcoma cell lines.** (**A**) Heat map illustrating hierarchical clustering of 36 differential miRNAs in UOSR2 and paired U2OS. (**B**) Volcano plot of differential miRNAs. Up-regulated miRNAs (red) and down-regulated miRNAs (green) were determined under the conditions of fold change (U2OSR2 versus U2OS) and false discovery rate-adjusted p-value ≤ 0.05. P-value was transformed by −log10. The fold change was also log-transformed. (**C**) Expression of microRNAs in U2OS after doxorubicin stimulation. (**D**–**E**) Western blot analysis of PD-L1 in U2OS with stimulation of different concentration of doxorubicin. (**F**–**G**) Western blot analysis of PD-L1 in K7 with stimulation of different concentration of doxorubicin. (**H**) qRT-PCR analysis of PD-L1 in U2OS with stimulation of different concentration of doxorubicin. (**I**) qRT-PCR analysis of PD-L1 in K7 with stimulation of different concentration of doxorubicin. (**J**) qRT-PCR analysis of miR-200a in U2OS with stimulation of different concentration of doxorubicin. (**K**) qRT-PCR analysis of miR-200a in K7 with stimulation of different concentration of doxorubicin. *P<0.05, **P<0.01, ***P<0.001.

To screen for the miRNAs involved in the regulation of PD-L1 in osteosarcoma, we performed miRNA-sequencing (miR-seq) on U2OSR2 and U2OS cells (Figure 1A, 1B). We initially identified 36 differentially expressed miRNAs using a 4-fold change cutoff and P<0.05 (Supplementary Table 1). Then, 10 miRNAs were selected from the differentially expressed miRNAs based on P values for additional detection in doxorubicin-treated osteosarcoma cells (1.6 μM for 24h) (Table 1, Figure 1C). Among them, the change of miR-200a was the most obvious, and we carried out further experiments especially for miR-200a. We observed a dose-dependent increase in the expression of PD-L1 (Figure1D–1I) and miR-200a (Figure 1J, 1K) in doxorubicin-treated U2OS and K7 cells. These results suggest that miR-200a regulates PD-L1expression in doxorubicin-treated osteosarcoma cells.

**Table 1:** Selected miRNAs from RNA-seq analyses of U2OSR2 and U2OS.

**Gene**	**Fold change (U2OSR2-U2OS)**	**P values**
hsa-miR-429	9.620479564	0.0001
hsa-miR-1269a	-8.569039824	0.0012
hsa-miR-3616-5p	-8.252257342	0.0023
hsa-miR-891a-5p	6.217150707	0.0023
hsa-miR-7854-3p	-5.33468257	0.0026
hsa-miR-200a	6.704126303	0.0036
hsa-miR-3689e	9.49619144	0.0071
hsa-miR-3689a-5p	9.247935763	0.0082
hsa-miR-3157-5p	7.851182879	0.0114
hsa-miR-3149	-4.62892541	0.0128

### miRNA-200a overexpression upregulates PD-L1 in osteosarcoma cells

**Figure 2:**
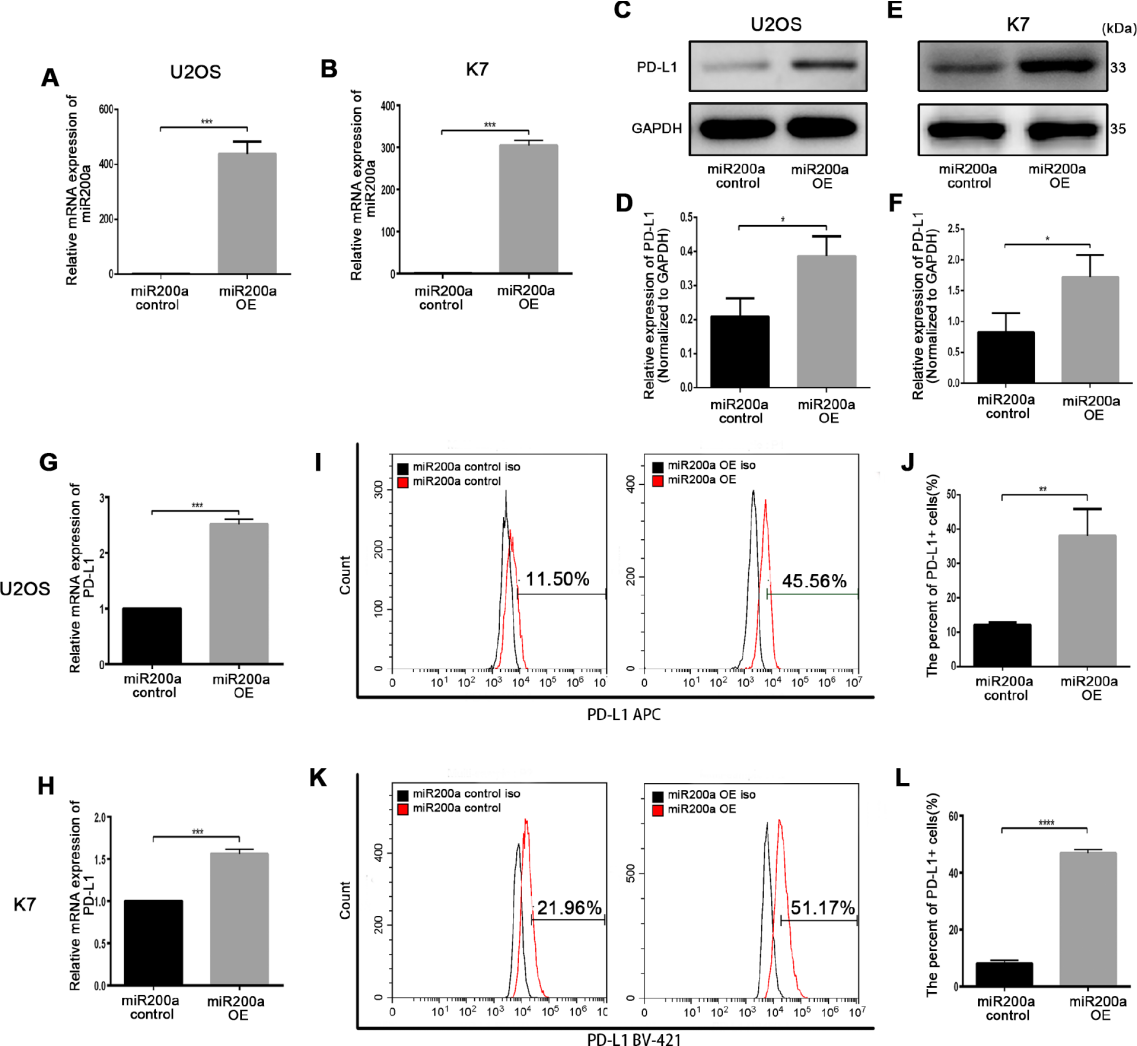
**miR-200a up-regulated PD-L1 expression in U2OS and K7.** (**A**) qRT-PCR analysis of miR-200a in U2OS miR-200a control and miR-200a OE. (**B**) qRT-PCR analysis of miR-200a in K7 miR-200a control and miR-200a OE. (**C**–**D**) Western blot analysis of PD-L1 in U2OS miR-200a control and miR-200a OE. (**E**–**F**) Western blot analysis of PD-L1 in K7 miR-200a control and miR-200a OE. (**G**) qRT-PCR analysis of PD-L1 in U2OS miR-200a control and miR-200a OE. (**H**) qRT-PCR analysis of PD-L1 in K7 miR-200a control and miR-200a OE. (**I**–**J**) Flow cytometry analysis of PD-L1 in U2OS miR-200a control and miR-200a OE. (**K**–**L**) Flow cytometry analysis of PD-L1 in K7 miR-200a control and miR-200a OE. *P<0.05, **P<0.01, ***P<0.001.

First, we investigated the role of miR-200a in osteosarcoma by analyzing the expression of miR-200a in human osteosarcoma cell lines, U2OS, HOS, MG63, 143B, and WELL5, and murine osteosarcoma cell lines, K7, K7M2, and DUNN. As shown in Supplementary Figure 1A, 1B, miR-200a levels were lowest in the U2OS and K7 cells, and highest in the 143B cells. Thus, we chose U2OS, K7, and 143B cells for further experiments. Next, to further study the relationship between miR-200a and PD-L1, we generated control and miR-200a-overexpressing osteosarcoma cells using lentiviral vectors called miR-200a control and miR-200a OE, respectively (Figure 2A, 2B). Western blot and quantitative RT-PCR (qRT-PCR) analysis showed significantly higher expression of PD-L1 in the miR-200a OE U2OS and K7 cells compared to the corresponding controls (Figure 2C–2H). Moreover, flow cytometry analysis also showed higher cell surface expression of PD-L1 in miR-200a OE U2OS and K7 cells compared to the corresponding controls (Figure 2I–2L). The miR-200a OE 143B cells also showed significantly higher expression of PD-L1 compared to the controls (Supplementary Figure 2A–2F). These data show that miR-200a overexpression does upregulate PD-L1 expression in osteosarcoma cells.

### miRNA-200a inhibits the activation and function of CD8^+^ T cells by upregulating PD-L1 *in vitro*

**Figure 3:**
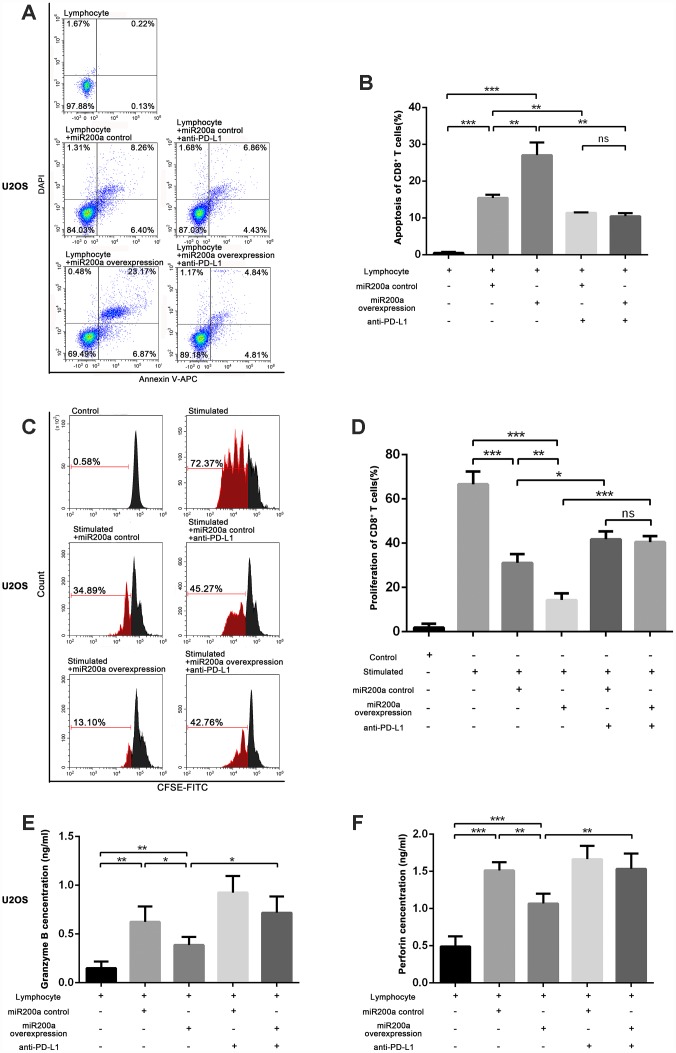
**miR-200a inhibited the function of CD8^+^ T cells through PD-L1/PD-1 pathway *in vitro*.** (**A**–**B**) Examine the apoptosis of CD8^+^ T cells after co-cultured with U2OS miR-200a control or miR-200a OE separately. (**C**–**D**) Examine the proliferation rate of CD8^+^ T cells after co-cultured with U2OS miR-200a control or miR-200a OE separately. (**E**–**F**) Enzyme-Linked ImmunoSorbent Assay (ELISA) of the secretion of granzyme B and perforin. *P<0.05, **P<0.01, ***P<0.001.

To explore the effects of miR-200a on anti-tumor immunity, we examined the functional status of CD8^+^ T cells after co-culturing with control and miR-200a OE osteosarcoma cells. CD8^+^ T cells co-cultured with miR-200a OE U2OS cells showed significantly higher apoptosis compared to the controls (Figure 3A–3B). The same phenomenon was also noted in 143B and K7 cells (Supplementary Figure 3A–3D).

We then analyzed the proliferation status of CD8^+^ T cells when co-cultured with control and miR-200a OE osteosarcoma cells. There was an increased proliferation rate of CD8^+^ T cells after the stimulation of CD3 and CD28 (Figure 3C, 3D). However, the proliferation rate of CD8^+^ T cells was significantly lower when co-cultured with miR-200a control U2OS, 143B, K7 cells, and lowest when co-cultured with miR-200a OE U2OS, 143B, K7 cells (Figure 3C, 3D; Supplementary Figure 4A–4D).

Furthermore, we analyzed the secretory function of CD8^+^ T cells by estimating the concentrations of secreted granzyme B and perforin. We observed that the concentrations of granzyme B and perforin were markedly decreased after co-cultured with miR-200a U2OS OE cells compared to the controls (Figure 3E, 3F).

We then evaluated whether immunosuppression induced by miR-200a was related to the upregulation of PD-L1 via adding anti-PD-L1 antibody during co-culture *in vitro*. We observed that the functions of CD8^+^ T cells were partially restored when co-cultured with U2OS, 143B and K7 cells in the presence of anti-PD-L1 antibody, but was still lower than CD8^+^ T cells cultured alone (Figure 3A–3F; Supplementary Figures 3A–3D, 4A–4D). These data suggest that miR-200a suppresses the function of CD8^+^ T cells and promotes the survival of osteosarcoma cells *in vitro*.

### miRNA-200a promotes the growth of osteosarcoma by impairing anti-tumor immunity via PD-L1 *in vivo*

**Figure 4:**
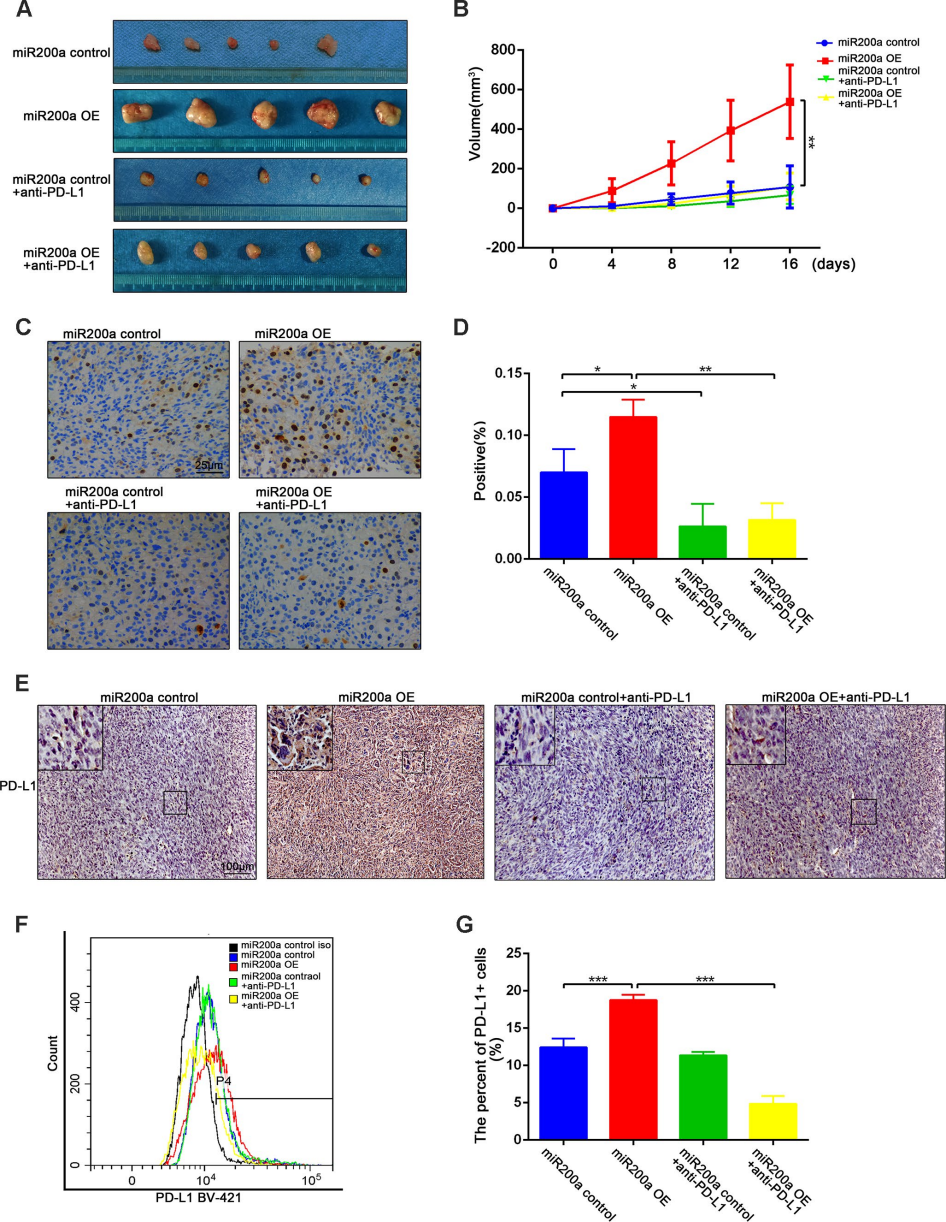
**miR-200a promoted tumor growth by up-regulating the expression of PD-L1 *in vivo*.** (**A**) miR-200a promoted the growth of osteosarcoma through up-regulating PD-L1. (**B**) Quantification of tumor volume. (**C**–**D**) Immunohistochemical staining analysis of ki-67 expression in tumor tissues. Scale bar represents 50μm. (**E**) Immunohistochemical staining analysis of PD-L1 expression in tumor tissues. Scale bar represents 100μm. (**F**–**G**) Flow cytometry analysis of PD-L1 expression in tumor tissues. *P<0.05, **P<0.01, ***P<0.001.

We used the K7-derived syngeneic mouse model to analyze the role of miR-200a in osteosarcoma *in vivo*. As shown in Figure 4A, 4B, the tumor volume of miR-200a-overexpressing K7 cells (miR-200a OE group) was significantly larger than that of the control group. Furthermore, the positive rate of ki-67 was significantly higher in miR-200a OE group compared to the controls (Figure 4C, 4D). This suggests that miR-200a promotes tumorigenesis partly by increasing the viability of osteosarcoma cells. Moreover, the overexpression of miR-200a could upregulate the expression of PD-L1 continuously *in vivo* as the expression of PD-L1 in the miR-200a OE group was significantly higher compared to the controls (Figure 4E–4G).

**Figure 5:**
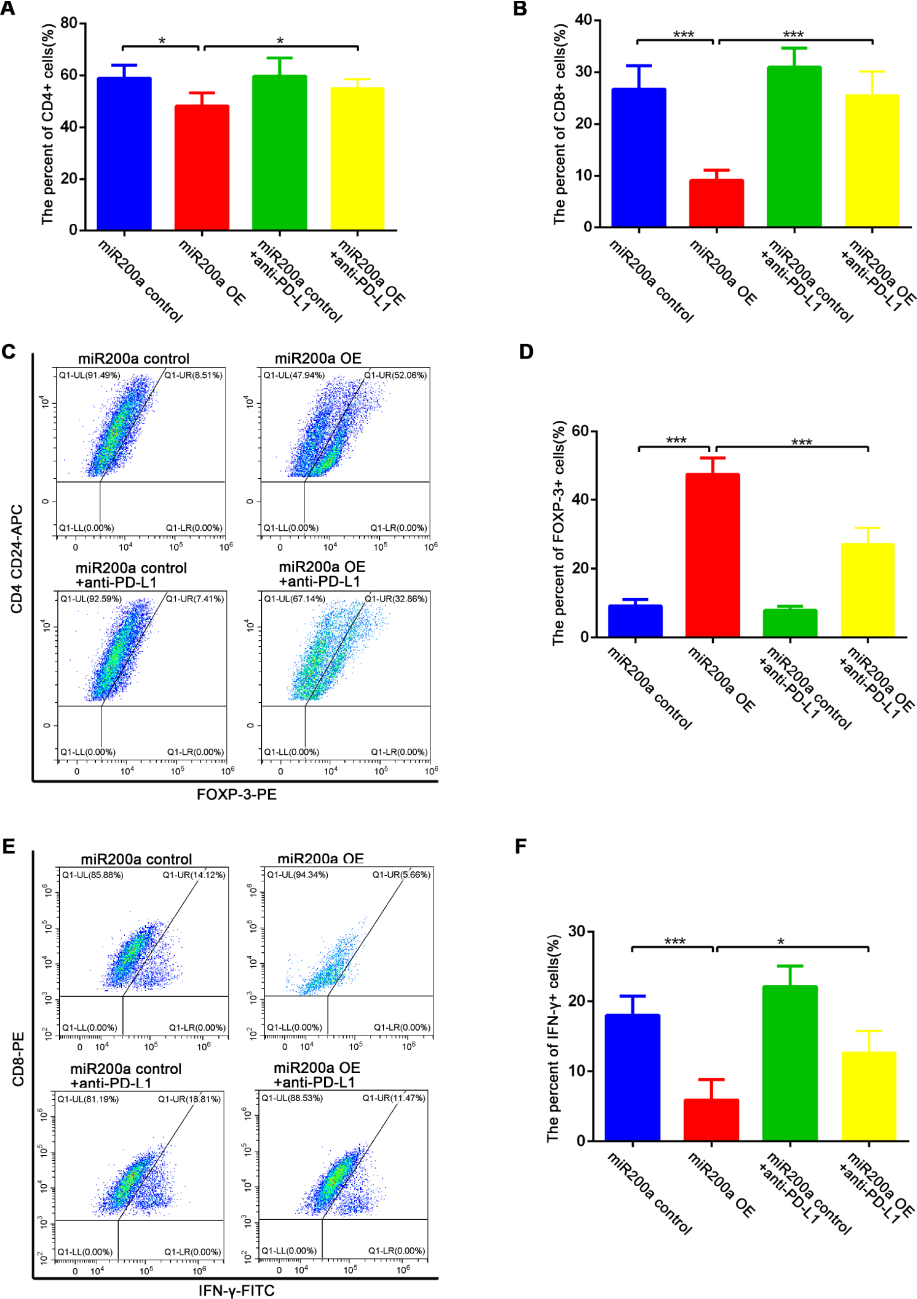
**miR-200a impaired anti-tumor immunity *in vivo*.** (**A**–**B**) Flow cytometry analysis of intratumoral proportion of CD4^+^ and CD8^+^ T cells. (**C**–**D**) Flow cytometry analysis of intratumoral proportion of Foxp3^+^ Treg cells. (**E**–**F**) Flow cytometry analysis of intratumoral proportion of IFN-γ^+^ CTLs. *P<0.05, **P<0.01, ***P<0.001.

We further examined the proportions of CD4^+^ T cells, CD8^+^ T cells, Foxp3^+^ regulatory T lymphocytes (T-regs), and IFN-γ^+^ cytotoxic T lymphocytes (CTLs) in the different groups of tumor tissues to determine the effects of miR-200a on anti-tumor immunity *in vivo*. The decreased proportions of intra-tumoral CD4^+^ and CD8^+^ T cells were observed in miR-200a OE group compared to the controls, for which the decrease in the proportion of CD8^+^ T cells was more obvious (Figure 5A, 5B). Moreover, the percentage of Foxp3^+^ T-regs (Figure 5C–5D) was significantly higher and the percentage of IFN-γ^+^ CTLs (Figure 5E–5F) was significantly lower in the miR-200a OE group compared to the control group. However, treatment with the anti-PD-L1 antibody significantly restored the anti-tumor immunity in the miR-200a OE group (Figures 4A–4D, 5A–5F). These data demonstrate that miR-200a promotes the growth of osteosarcoma by impairing anti-tumor immunity *in vivo*, but at the same time, miR-200a OE group is also more sensitive to the treatment of anti-PD-L1 antibodies.

### High expression of miR-200a predicts the poor response to chemotherapy in osteosarcoma patients

**Figure 6:**
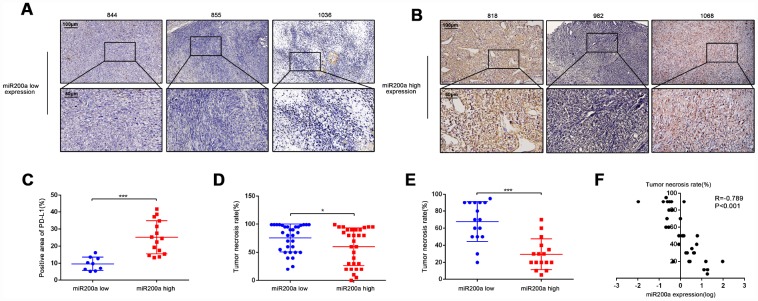
**High expression of miR-200a predicts the poor response to chemotherapy.** (**A**–**C**) Immunohistochemical staining analysis of PD-L1 expression in samples of osteosarcoma patients. The quantification of immunohistochemical staining was based on its positive area. Scale bar represents 50μm and 100μm separately. (**D**) Comparison of TNR in biopsy tissue of 62 osteosarcoma patients acquired from the GEO database (GSE39058-GPL15762). Divided them into two groups according to miR-200a expression. (**E**) Comparison of TNR in resection tissues of 32 osteosarcoma patients. Divided them into two groups according to miR-200a expression. (**F**) Correlation analysis between miR-200a and TNR in resection tissues of 32 osteosarcoma patients. The expression of miR-200a was log-transformed. *P<0.05, **P<0.01, ***P<0.001.

We performed qRT-PCR and immunohistochemical analysis of the tumor specimens from 32 osteosarcoma patients to verify the relationship between miR-200a and PD-L1 (Table 2). The patients were divided into high- and low- miR-200a expression groups. As expected, patients with high miR-200a expression showed higher PD-L1 positivity than patients with low miR-200a expression (Figure 6A–6C). Our previous research showed that the therapeutic efficacy of doxorubicin was reduced due to the doxorubicin-induced-upregulation of PD-L1 expression in osteosarcoma [3]. Therefore, we tested if the expression of miR-200a was related to the efficacy of chemotherapy in osteosarcoma by analyzing biopsy tissues from a cohort of 62 osteosarcoma patients from the GEO database (GSE39058-GPL15762). The tumor necrosis rate (TNR) after conventional chemotherapy was significantly lower in osteosarcoma patients with high miR-200a expression compared to patients with lower miR-200a expression (Figure 6D; 60.10% vs. 75.59%; P=0.0407). Similar results were not observed for miR-200b and miR-200c (data not shown). Subsequently, we validated these results using fresh resection samples from 32 osteosarcoma patients (Figure 6E; 29.38% vs 67.81%; P < 0.001). Furthermore, the correlation analysis proposed to a negative correlation between miR-200a expression and TNR (Figure 6F; R=-0.789; P<0.001). Our results indicate that elevated expression of miR-200a is associated with increased expression of PD-L1 and predicts a poor response to chemotherapy.

**Table 2:** Patient information.

**No. Patients**	**Sex**	**Age (years)**	**Location of primary tumor**	**Relative expression of miR-200a**	**Tumor necrosis rate (%)**	**PD-L1 (%)**
724	M	6	Femur	1	70	16.247
731	F	38	Femur	2.06893	30	NA
778	M	16	Femur	0.84761	50	NA
798	F	62	Tibia	0.30743	90	6.452
818	F	17	Femur	12.73857	10	35.649
822	M	28	Femur	0.24555	90	12.783
827	F	33	Femur	18.09060	20	41.708
841	F	52	Femur	0.15701	90	NA
844	M	13	Femur	0.01051	90	9.832
855	F	12	Tibia	0.23429	70	5.11
874	M	16	Femur	94.81453	20	38.602
881	F	38	Femur	0.29056	80	NA
885	M	57	Pelvis	0.46572	20	NA
892	M	13	Fibula	5.79038	40	37.126
930	M	19	Humerus	3.41790	35	13.712
947	F	7	Humerus	1.80640	30	19.37
982	M	16	Humerus	18.20056	5	31.539
986	M	21	Tibia	0.21040	60	5.3
1014	M	10	Humerus	0.21226	95	13.652
1036	F	17	Femur	0.38472	90	7.014
1062	M	6	Humerus	6.85090	60	34.712
1065	M	13	Femur	0.28123	60	10.373
1068	F	17	Femur	1.23025	50	25.297
1074	M	14	Humerus	4.17052	30	24.5
1079	M	9	Femur	2.78765	20	NA
1096	M	17	Tibia	1.43837	50	15.428
1099	M	16	Femur	18.63667	10	22.417
1114	M	15	Femur	0.85041	40	NA
1126	M	18	Humerus	0.38215	90	NA
1180	F	28	Pelvis	4.44639	30	13.372
1182	M	18	Femur	4.32866	30	17.528
1201	M	13	Tibia	1.47352	90	NA

### miRNA-200a induces PD-L1 expression by direct targeting PTEN

**Figure 7:**
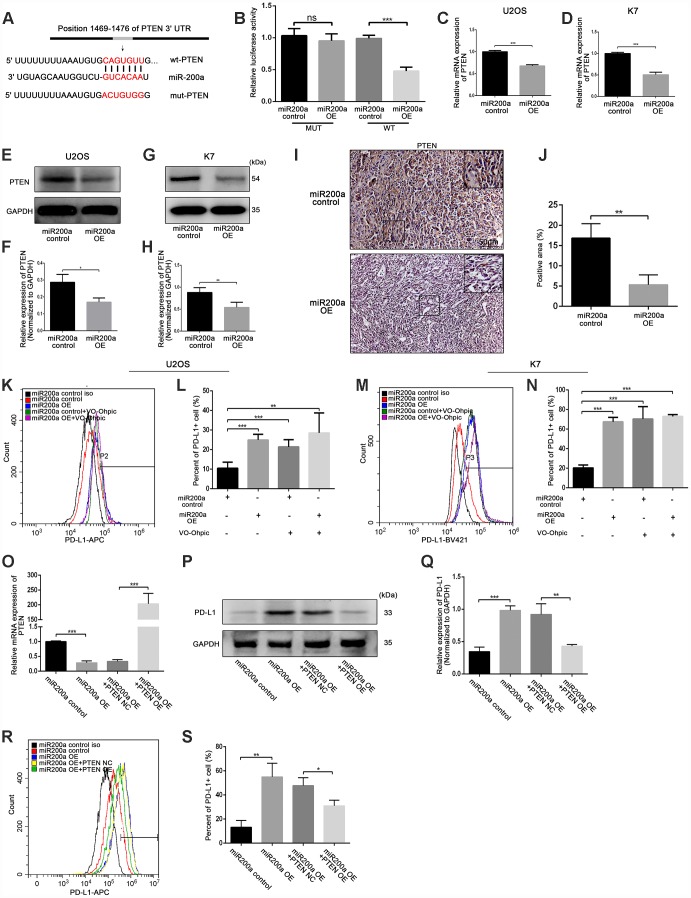
**miR-200a up-regulated PD-L1 expression by targeting PTEN.** (**A**) miR-200a target sequence binding to PTEN 3-UTR was predicted with TargetScan. mut-PTEN, mutated from the seed matches, was indicated. (**B**) Transfected miR-200a overexpressing plasmid with wt-PTEN or mut- PTEN separately in U2OS, measured with luciferase assays. A Renilla luciferase plasmid was co-transfected as a transfection control. (**C**) qRT-PCR analysis of PTEN in U2OS miR-200a control and miR-200a OE. (**D**) qRT-PCR analysis of PTEN in K7 miR-200a control and miR-200a OE. (**E** and **F**) Western blot analysis of PD-L1 in U2OS miR-200a control and miR-200a OE. (**G** and **H**) Western blot analysis of PD-L1 in K7 miR-200a control and miR-200a OE. (**I** and **J**) Immunohistochemical staining analysis of PTEN expression in tumor tissues. Scale bar represents 50μm. (**K** and **L**) Flow cytometry analysis of PD-L1 expression of U2OS miR-200a control and miR-200a OE after the addition of VO-Ohpic. (**M** and **N**) Flow cytometry analysis of PD-L1 expression of K7 miR-200a control and miR-200a OE after the addition of VO-Ohpic. (**O**) qRT-PCR analysis of PTEN in U2OS miR-200a control and miR-200a OE after PTEN overexpressing. (**P** and **Q**) Western blot analysis of PD-L1 in U2OS miR-200a control and miR-200a OE after PTEN overexpressing. (**R** and **S**) Flow cytometry analysis of PD-L1 expression of U2OS miR-200a control and miR-200a OE after PTEN overexpressing. *P<0.05, **P<0.01, ***P<0.001.

To investigate the molecular mechanism by which miR-200a induced upregulation of PD-L1, we identified potential targets of miR-200a using Targetscan (http://www.targetscan.org). According to the prediction of Targetscan, PTEN was found as the target gene of miR-200a. The interaction between miR-200a and PTEN 3’-UTR in U2OS cells was analyzed using the dual-luciferase reporter assay. As shown in Figure 7A, 7B, there was a direct binding between PTEN 3’-UTR and miR-200a as the luciferase activity of the wild type-PTEN (wt-PTEN), but not the mutant-PTEN (mut-PTEN) was suppressed by the overexpression of miR-200a. We also observed that the expression of PTEN in miR-200a OE U2OS and K7 cells was significantly reduced compared to the controls (Figure 7C–7H).

Furthermore, the expression of PTEN was significantly lower in tumor tissues from the miR-200a OE group compared to the miR-200a control group *in vivo* (Figure 7I, 7J). Moreover, miR-200a control U2OS and K7 cells treated with VO-Ohpic, a PTEN selective inhibitor, showed increased expression of PD-L1 as analyzed by flow cytometry (Figure 7K–7N). However, miR-200 OE U2OS and K7 cells did not further increase the already elevated expression of PD-L1 when treated with VO-Ohpic (Figure 7K–7N).

To further confirm the role of PTEN in miR-200a-induced upregulation of PD-L1, we constructed a stable PTEN overexpression cell line by lentiviral transfection in U2OS cells (Figure 7O). As shown in Figure 7P–7S, the overexpression of PTEN significantly attenuated the upregulation of PD-L1 induced by miR-200a overexpression. And we also showed that PTEN acted as the target gene of miR-200a in 143B cells (Supplementary Figure 5A–5E).

Taken together, our results demonstrate that miR-200a upregulates the expression of PD-L1 by binding to PTEN 3’-UTR directly. Furthermore, miR-200a overexpression promotes the growth of osteosarcoma by inducing immunosuppression via PD-L1. And the high expression of miR-200a predicts a better response to PD-L1-targeted immunotherapy and a poorer response to chemotherapy in osteosarcoma.

## DISCUSSION

**Figure 8:**
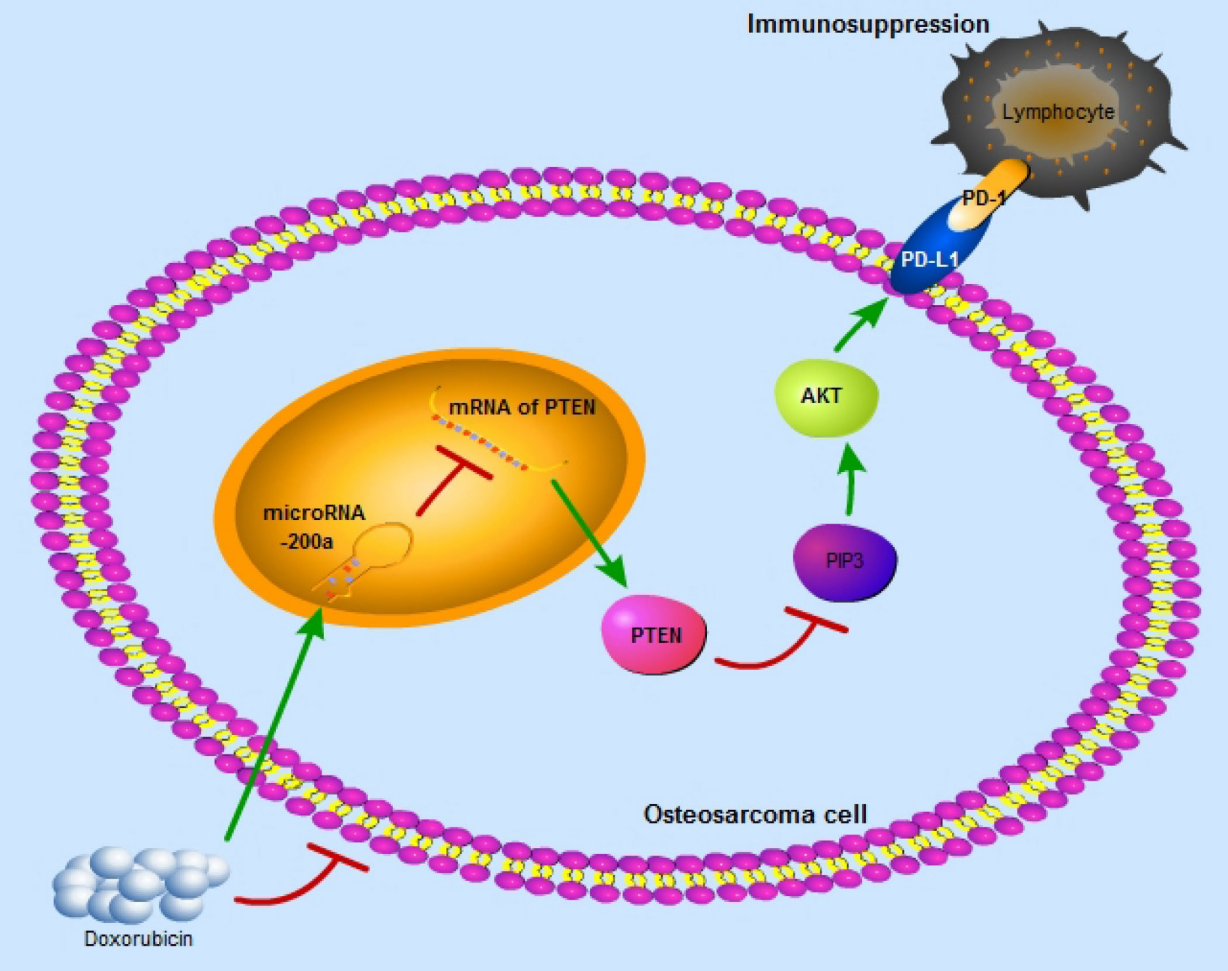
**The schematic graph reflects miR-200a/PTEN/PD-L1 axis in the osteosarcoma cells to induce immunosuppression.**

Recent studies have suggested that PD-L1-targeted immunotherapy is a potential treatment strategy for osteosarcoma patients. However, the clinical response to this kind of immunotherapy is poor even in cases with high expression of PD-L1. Therefore, it is imperative to clarify the regulatory mechanism of PD-L1 and improve the efficacy of PD-L1-targeted immunotherapy in osteosarcoma. In this study, we propose that miR-200a upregulates the expression of PD-L1 by binding to PTEN and impairs the anti-tumor immunity to promote tumor growth. We also demonstrate that higher miR-200a levels are associated with lower TNR after conventional chemotherapy in osteosarcoma. Moreover, high miR-200a expression may predict a better response to PD-L1-targeted immunotherapy in osteosarcoma. In conclusion, our study demonstrates that the miR-200a/PTEN/PD-L1 axis regulates osteosarcoma growth and response to chemotherapy and PD-L1-targeted immunotherapy (Figure 8).

High expression of PD-L1 is correlated with poor clinical prognosis in several tumors [17, 18]. Ligation of PD-L1 on cancer cells to PD-1 on T cells suppressed T cells activation and proliferation and induced T cells apoptosis [19, 20]. Previously, we observed that doxorubicin treatment induced the immunosuppression of CD8^+^ T cells by upregulating PD-L1 in osteosarcoma [3]. miRNA-seq analysis showed increased expression of miR-200a in the U2OSR2 cells compared to the U2OS cells. We also observed that doxorubicin treatment increased miR-200a levels in U2OS and K7 cells. These findings suggest that miR-200a may regulate PD-L1 expression in osteosarcoma cells in response to chemotherapy. As expected, miR-200a overexpression induced PD-L1 expression in the osteosarcoma cells. These results are contrary to findings in breast cancers and NSCLC, in which the miR-200 family members inhibit the expression of PD-L1 [21, 22] and act as tumor suppressors [23]. Thus, to verify the reliability of the lentiviral we constructed to carry miR-200a, we transfected MCF7 cells with the same lentivirus and obtained similar results as reported previously (Supplementary Figure 6A–6F). The explanation for the divergent results may due to the heterogeneity of tumors. Osteosarcoma is derived from mesenchymal cells, whereas breast cancer, as well as NSCLC are derived from epithelial cells. Previous studies have shown that the miR-200 family members inhibit epithelial-mesenchymal transition (EMT) and promote the mesenchymal-epithelial transition (MET) by binding to ZEB1 [24] or Sec23a [25]. Since osteosarcoma is a mesenchyme-derived malignancy, the inhibitory effect of miR-200 family on EMT may be less important to osteosarcoma. Furthermore, Xu *et al* and Liu *et al* reported that miR-200 family members acted as tumor suppressors and inhibit osteosarcoma cell proliferation [26, 27]. The same miRNA performs pleiotropic functions during tumorigenesis and development because the seed sequence of miRNAs can bind to multiple transcripts [28]. The previous studies mentioned above mainly focused on the effects of the miR-200 family members on the osteosarcoma cells *in vitro*, without considering the complexity of tumor-immune interactions that are relevant *in vivo*. In our study, we performed experiments to explore the effects of miR-200a on anti-tumor immunity in osteosarcoma both *in vitro* and *in vivo.* Although the positive rate of ki-67 in miR-200a OE group was significantly higher than that of miR-200a control group in xenograft experiments (10.88% vs 5.34%, P=0.0012), *in vitro* experiments did not show any significant differences in viability between the control and miR-200a OE K7 cells (Supplementary Figure 7). This further suggests that miR-200a promotes the growth of osteosarcoma cells mainly through impairing anti-tumor immunity. However, the osteosarcoma cells were not in contact with the lymphocytes when cultured *in vitro*, and hence, we did not observe viability differences between the two groups. Therefore, these data suggest that miR-200a affects osteosarcoma cell viability indirectly by modulating anti-tumor immunity.

We also explored the effect of miR-200a on anti-tumor immunity in this study. We focused on CD8^+^ T cells in this study as they are the main effector cells of anti-tumor immunity and are essential for the efficacy of immunotherapy against PD-L1 [29, 30]. We examined the effect of miR-200a on apoptosis and proliferation of CD8^+^ T cells *in vitro* and found that miR-200a induced apoptosis and inhibited proliferation of CD8^+^ T cells. In addition, miR-200a overexpression in osteosarcoma cells also inhibited the secretory function of CD8^+^ T cells. Moreover, the immunosuppression induced by miR-200a was closely related to the upregulation of PD-L1, as the treatment with anti-PD-L1 antibodies could partially reverse the immunosuppressive effects of osteosarcoma cells on CD8^+^ T cells.

*In vivo* experiments showed that xenografted miR-200a OE K7 cells generated larger tumors by inhibiting anti-tumor immunity. The miR-200a OE group showed decreased proportions of CD4^+^T cells, CD8^+^ T cells and IFN-γ^+^ CTLs and an increased percentage of Foxp3^+^ T-regs in tumors. Although miR-200a promoted the growth of osteosarcoma *in vivo*, the anti-tumor effect of anti-PD-L1 antibodies was brilliant in miR-200a OE group. As the study by Wei et al. indicated that cancer hallmarks of human PD-L1^+^ tumors determine the therapeutic efficacy of PD-L1-targeted immunotherapy [31]. And our study indicates that high expression of miR-200a may be a kind of cancer hallmarks which indicates a better response to PD-L1-targeted immunotherapy in osteosarcoma.

We also examined the expression of miR-200a and PD-L1 in the samples of osteosarcoma patients to further verify our results and explore the clinical implications of our findings. We observed that patients with high miR-200a levels showed an increased positive rate of PD-L1, poor response to chemotherapy, and low TNR. Therefore, as shown by Li et al. for miR-423 in glioblastomas [32], we postulate that therapeutically targeting miR-200a in combination with conventional chemotherapy may improve the survival rate of osteosarcoma patients. However, this hypothesis needs to be further investigated.

Furthermore, we explored the potential mechanism by which miR-200a regulated expression of PD-L1. We identified PTEN as one of the target genes of miR-200a. It has been reported that PTEN inhibited the expression of PD-L1 in lung squamous cell carcinoma and colorectal cancer by inhibiting the PI3K/Akt pathway [33, 34]. Previous studies also showed that microRNA-200c acted as an oncogene in nasopharyngeal carcinoma by targeting PTEN [35]. Consistent with these studies, our results demonstrate that miR-200a upregulates the expression of PD-L1 by binding to the PTEN 3’UTR and inhibiting its expression in osteosarcoma.

In conclusion, our study proposes a unique regulatory pathway, the miR-200a/PTEN/PD-L1 axis, which is related to the growth of osteosarcoma and its response to PD-L1-targeted immunotherapy.

## MATERIALS AND METHODS

### Osteosarcoma patient surgical specimens

Samples from 32 osteosarcoma patients (11 females, 21 males) of ages 6 to 62 years (mean = 21.13 years) were selected for RNA extraction. 24 of the 32 samples were chosen for immunohistochemical staining of PD-L1. Tumor necrosis rate (TNR) was judged by 3 independent pathologists from the pathology department of Ruijin Hospital, Shanghai, China. In the correlation analysis between miR-200a and TNR, we performed a log transformation of miR-200a expression. General information on all 32 patients was presented in Table 2. This study was approved by the Ethics Committee of Ruijin Hospital, affiliated with Shanghai Jiaotong University School of Medicine.

### Quantitative RT-PCR analysis

mRNA expression of miRNAs, PD-L1, and PTEN was analyzed by qRT-PCR as previously described [36]. All primer sequences were presented in Table 3. U6 or GAPDH served as the reference gene, and the expression of target genes was calculated using 2^—ΔΔCT^.

**Table 3:** Primer sequence.

**Gene**	**Primer sequence (5’-3’)**
**U6**	Forward:CGCTTCGGCAGCACATATAC
	Reverse:AAATATGGAACGCTTCACGA
**miR-200a**	Forward:GCGCGTAACACTGTCTGGTAA
	Reverse:AGTGCAGGGTCCGAGGTATT
**miR-200b**	Forward:GCGCATCTTACTGGGCAGC
	Reverse:AGTGCAGGGTCCGAGGTATT
**miR-200c**	Forward:GCGCGTCTTACCCAGCAGT
	Reverse:AGTGCAGGGTCCGAGGTATT
**GAPDH**	Forward:TGTGGGCATCAATGGATTTGG
**(human)**	Reverse:ACACCATGTATTCCGGGTCAAT
**GAPDH**	Forward:TTGTCATGGGAGTGAACGAGA
**(mouse)**	Reverse:CAGGCAGTTGGTGGTACAGG
**PD-L1**	Forward:GCTGCACTAATTGTCTATTGGG
**(human)**	Reverse:CACAGTAATTCGCTTGTAGTCG
**PD-L1**	Forward:AAGCCTCAGCACAGCAACTTCAG
**(mouse)**	Reverse:TGTAGTCCGCACCACCGTAGC
**PTEN**	Forward:ATGTTCAGTGGCGGAACTTG
**(human)**	Reverse:CACACAGGTAACGGCTGAGG
**PTEN**	Forward:TGGATTCGACTTAGACTTGACCT
**(mouse)**	Reverse:GCGGTGTCATAATGTCTCTCAG

### Immunohistochemistry

Immunohistochemical staining of PD-L1, PTEN, and ki-67 was performed using the Super Sensitive IHC Detection System Kit (BD5001, Bioworld) according to the manufacturer’s instruction. Slides were incubated with antibodies against PD-L1 (10084-MB55, Sino biological, China), PTEN (9188, CST, USA) and ki-67 (ab16667, Abcam, USA) overnight at 4°C. Slides were evaluated by 3 independent investigators who were blinded to the identity of each slide. The positive rate of PD-L1 and ki-67 was quantified by Image J.

### Cell culture

Human osteosarcoma cell lines 143B, MG63, HOS, and U2OS cells were obtained from ATCC (Manassas, VA, USA), human osteosarcoma multidrug resistance (MDR) cell line U2OSR2 cells [37] and mouse osteosarcoma cell line K7, K7M2, and DUNN cells were kindly gifted by Dr. Zhengdong Cai of Shanghai General Hospital. All cells were cultured in high glucose DMEM supplemented with 10% fetal bovine serum and 1% penicillin-streptomycin at 37°C in 5% CO_2_. Different concentrations of doxorubicin (Sigma, USA) were added to the medium. VO-Ohpic (S8651, Selleck), a PTEN inhibitor, was added to the medium at 10 μM.

### Lentiviral vectors and transfection

Lentiviral vectors for miR-200a or PTEN overexpression were constructed by GeneChem (Shanghai, China). Both a recombinant lentivirus and negative control lentivirus (GFP-lentivirus) were prepared. 72 h after the viral infection, cells were treated with puromycin (GeneChem, China) at 5 μg/ml to construct cell lines stably overexpressing miR-200a (miR-200a OE) or PTEN (PTEN OE).

### microRNA sequencing (miRNA-seq)

RNA was isolated from U2OSR2 and U2OS cells using RNeasy kit (217004, Qiagen). miRNA-Seq libraries were performed using the Small RNA Library Prep (NEB E7300L/E7580L). miRNA-seq libraries were run on the HiSeq 2500 (Illumina) using Single-end 50-bp sequencing. miR-Seq toolbox with miRNA workflow was used. Briefly, reads were checked by FastQC v0.11.5 and aligned with Bowte v2.28. Differentially expressed genes (DEGs) analysis was performed with EdgeR. Data are presented as gene sequence fold-change in U2OSR2 cells versus U2OS cells.

### Cell viability measurement

K7 miR-200a control and miR-200a OE were seeded at a density of 5x10^3^ cells/well in 96-well plates. Cell counting kit-8 (CCK-8) (Dojindo, Japan) was used to measure the viability of osteosarcoma cells *in vitro* following the manufacturer’s instructions.

### Dual-luciferase reporter assay

Partial DNA sequences of PTEN 3’-UTR containing wild-type (wt) or mutant (mut) miR-200a binding sites were amplified by PCR and cloned into the GV272 reporter vector (GeneChem, China) to produce wt-PTEN and mut-PTEN reporter plasmids. Then, the constructed reporter plasmids and renilla luciferase reporter were co-transfected with miR-200a mimics or negative control into U2OS cells using lipofectamine 3000. 48h after transfection, luciferase activity was detected using a Dual-Luciferase Reporter Assay System (Promega, USA) and firefly luciferase units normalized against renilla luciferase units.

### Western blot analysis

Western blot was performed as previously described [38]. The blots were incubated with primary antibodies against PD-L1 (ab213524, Abcam, USA), PTEN (9188, CST, USA) and GAPDH (5174, CST, USA) overnight at 4°C. For proteins with similar molecular weights, we used Western Blot Fast Stripping Buffer (PS107, Epizyme, China) for 15 minutes and then incubated with another antibody. Protein amounts were determined by densitometric analysis and normalized to GAPDH levels.

### Flow cytometry

Osteosarcoma cells were digested by 0.25% trypsin with EDTA and incubated with anti-human PD-L1-APC (563741, BD bioscience, USA) or anti-mouse PD-L1-BV421 (564716, BD bioscience, USA) or isotype control antibodies for 30 min at room temperature in the dark. After incubation, cells were washed with phosphate buffered saline (PBS) then subjected to flow cytometry. When we analyzed the results, only osteosarcoma cells with GFP fluorescence were selected.

### CD8^+^ T cell proliferation assay

CD8^+^ T cell proliferation assay was performed as described previously [3]. The human anti-PD-L1 antibody (16-5983-82, eBioscience, USA) or mouse anti-PD-L1 antibody (16-5982-85, eBioscience, USA) was added to the medium at 5 μg/ml. After co-culture, we selected CD8^+^ T cell (CD3 and CD8 positive lymphocytes) to detect the proliferation of it by CytoFlex S (Beckman, USA).

### CD8^+^ T cell apoptosis assay

An Annexin V Apoptosis Detection Kit APC (88-8007-72, eBioscience, USA) was used to detect the apoptosis of CD8^+^ T cells. The detailed procedure was the same as described previously [3]. The binding of PD-L1 to PD-1 was blocked using the anti-PD-L1 antibody mentioned above. CD8^+^ T cell apoptosis was calculated as the percentage of annexin V^+^ cells in a gated CD8^+^ population.

### Cytokine assay

After co-culture, the supernatant from each well was collected and centrifugated to remove cell debris. Concentrations of granzyme B and perforin were then determined using an ELISA kit (BMS6029, Invitrogen, USA; BMS2306, Invitrogen, USA) according to the manufacturer’s introductions.

### Subcutaneous tumor models

The subcutaneous osteosarcoma models were performed as described previously using mouse osteosarcoma cell line K7 [3]. Mice were randomly divided into four groups: miR-200a control (with PBS only), miR-200a control (with anti-PD-L1 antibody), miR-200a OE (with PBS only) and miR-200a OE (with anti-PD-L1 antibody) (N = 5 per group). anti-PD-L1 antibody (BE0101, Bio X Cell, USA) was administered every 3 days intravenously at 10 mg/kg. Tumor size was measured every four days and calculated using the equation (length×width^2^) /2. This protocol was approved by the Ethics Committee of Ruijin Hospital, affiliated with Shanghai Jiaotong University School of Medicine.

### *In vivo* lymphocyte detection

16 days after implantation, tumors were dissected and lysed to a single cell suspension using type IV collagenase (1 mg/ml) and DNase I (0.2 mg/ml). The detailed procedure was the same as described previously [3]. The antibodies used were listed as below: PD-L1-BV421 (564716, BD bioscience, USA), CD3-APC (17-0032-82, eBioscience, USA), CD4-FITC (11-0041-82, eBioscience, USA), CD8-PE (12-0081-82, eBioscience, USA), Foxp3-PE (12-5773-82, eBioscience, USA), IFN-γ FITC (11-7311-82, eBioscience, USA).

### Statistical analysis

All data from three independent experiments are expressed as mean ± standard deviation. Statistical differences between groups were estimated using a Student's t-test or one-way ANOVA. Statistical analyses were performed using GraphPad Prism 5.0.

## Supplementary Material

Supplementary Figures

Supplementary Table 1
