# Supplementary material for: MicroRNA-200a induces immunosuppression by promoting PTEN-mediated PD-L1 upregulation in osteosarcoma
Source: Aging (Albany NY). 2020 Jan 24;12(2):1213–36. doi: 10.18632/aging.102679 (PMC7053609; doi:10.18632/aging.102679)
Supplement: Supplementary Figures [file aging-12-102679-s002..pdf]

SUPPLEMENTARY FIGURES

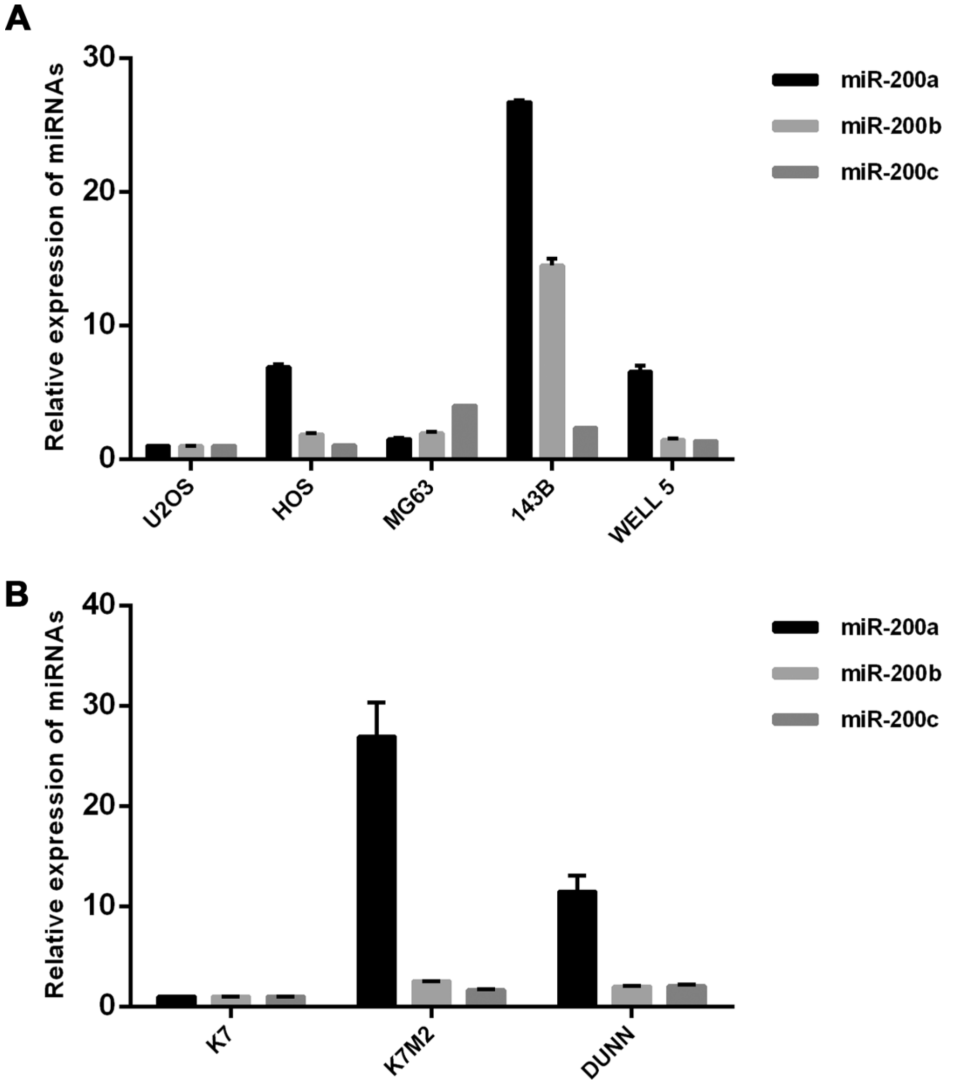

Supplementary Figure 1. Expression of miR-200 family in human and mouse osteosarcoma cell lines. (**A**) Expression of miR-200 family in human osteosarcoma cell lines. (**B**) Expression of miR-200 family in mouse osteosarcoma cell lines.

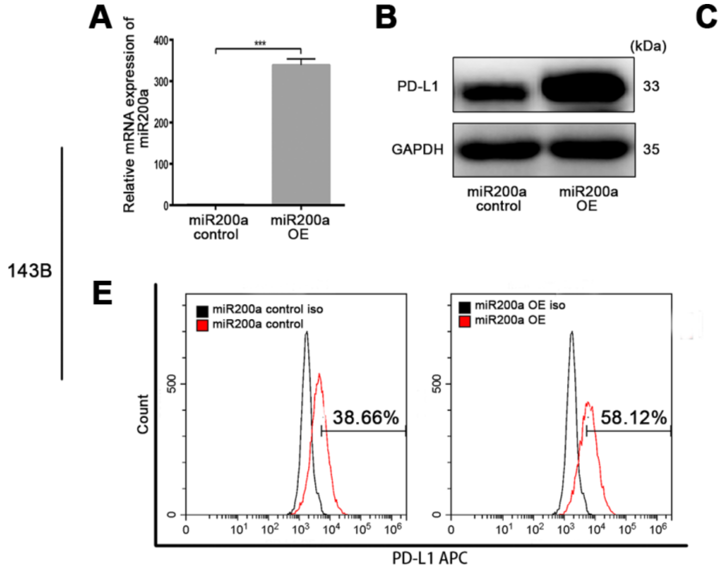

Supplementary Figure 2. miR-200a up-regulated PD-L1 expression in 143B. (**A**) qRT-PCR analysis of miR-200a in 143B miR-200a control and miR-200a OE. (**B**–**C**) Western blot analysis of PD-L1 in 143B miR-200a control and miR-200a OE. (**D**) qRT-PCR analysis of PD-L1 in 143B miR-200a control and miR-200a OE. (**E**–**F**) Flow cytometry analysis of PD-L1 in 143B miR-200a control and miR-200a OE. *P<0.05, **P<0.01, ***P<0.001.

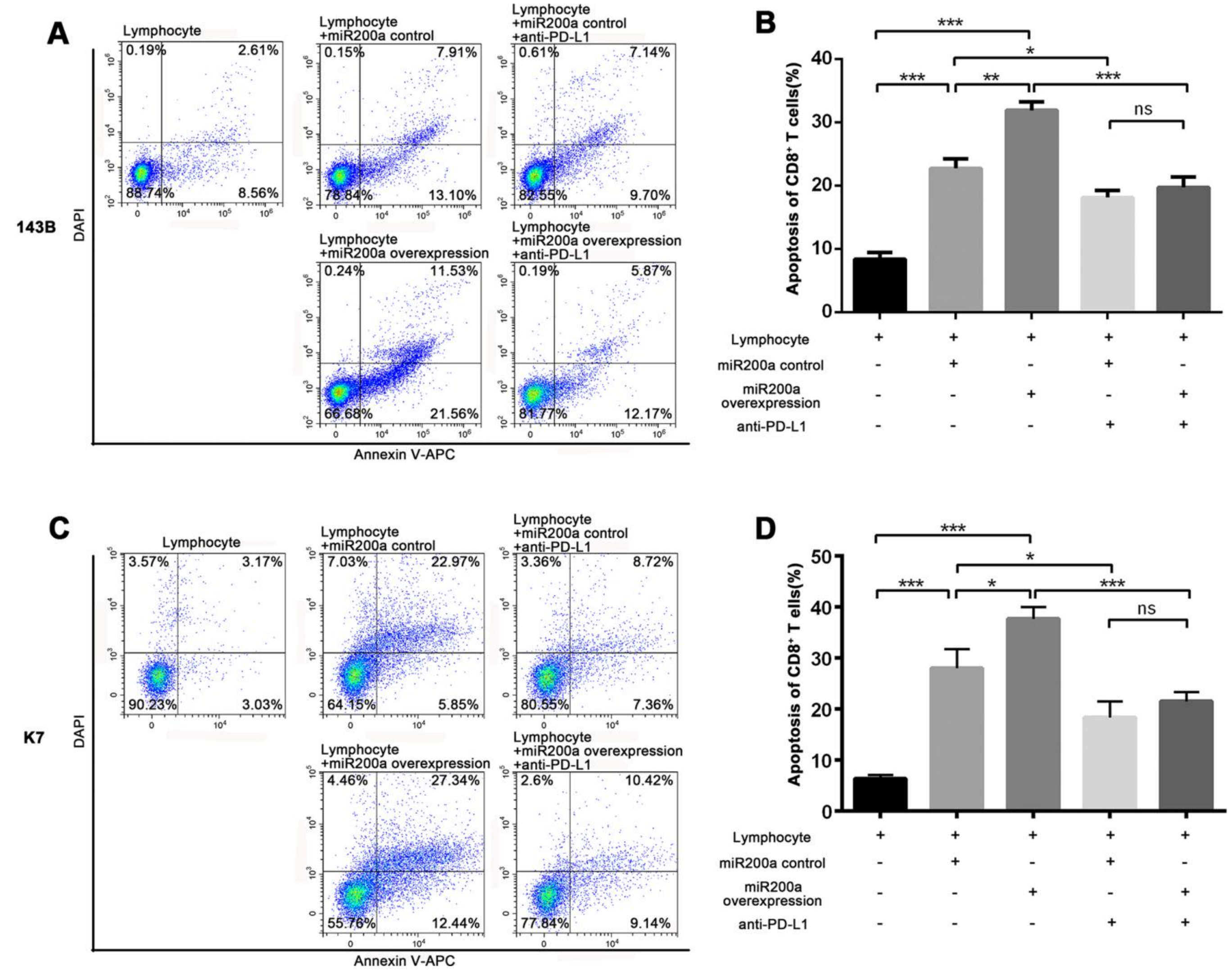

Supplementary Figure 3. miR-200a promoted the apoptosis of CD8$^+$ T cells through PD-L1/PD-1 pathway *in vitro*. (**A**–**B**) Examine the apoptosis of CD8$^+$ T cells after co-cultured with 143B miR-200a control or miR-200a OE. (**C**–**D**) Examine the apoptosis of CD8$^+$ T cells after co-cultured with K7 miR-200a control or miR-200a OE. *P<0.05, **P<0.01, ***P<0.001.

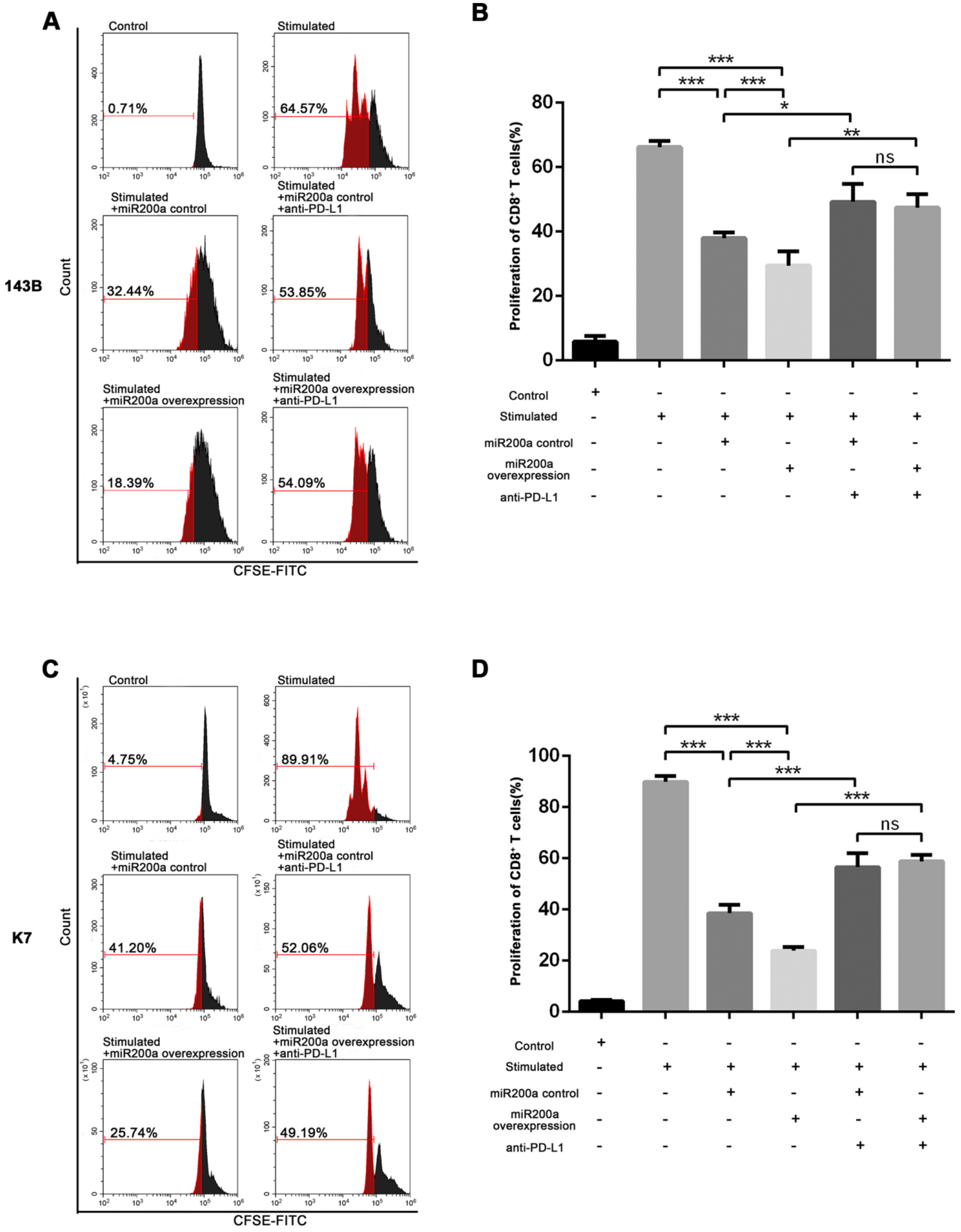

Supplementary Figure 4. miR-200a inhibited the proliferation of CD8$^+$ T cells through PD-L1/PD-1 pathway *in vitro*. (**A**–**B**) Examine the proliferation of CD8$^+$ T cells after co-cultured with 143B miR-200a control or miR-200a OE. (**C**–**D**) Examine the proliferation of CD8$^+$ T cells after co-cultured with K7 miR-200a control or miR-200a OE. *P<0.05, **P<0.01, ***P<0.001.

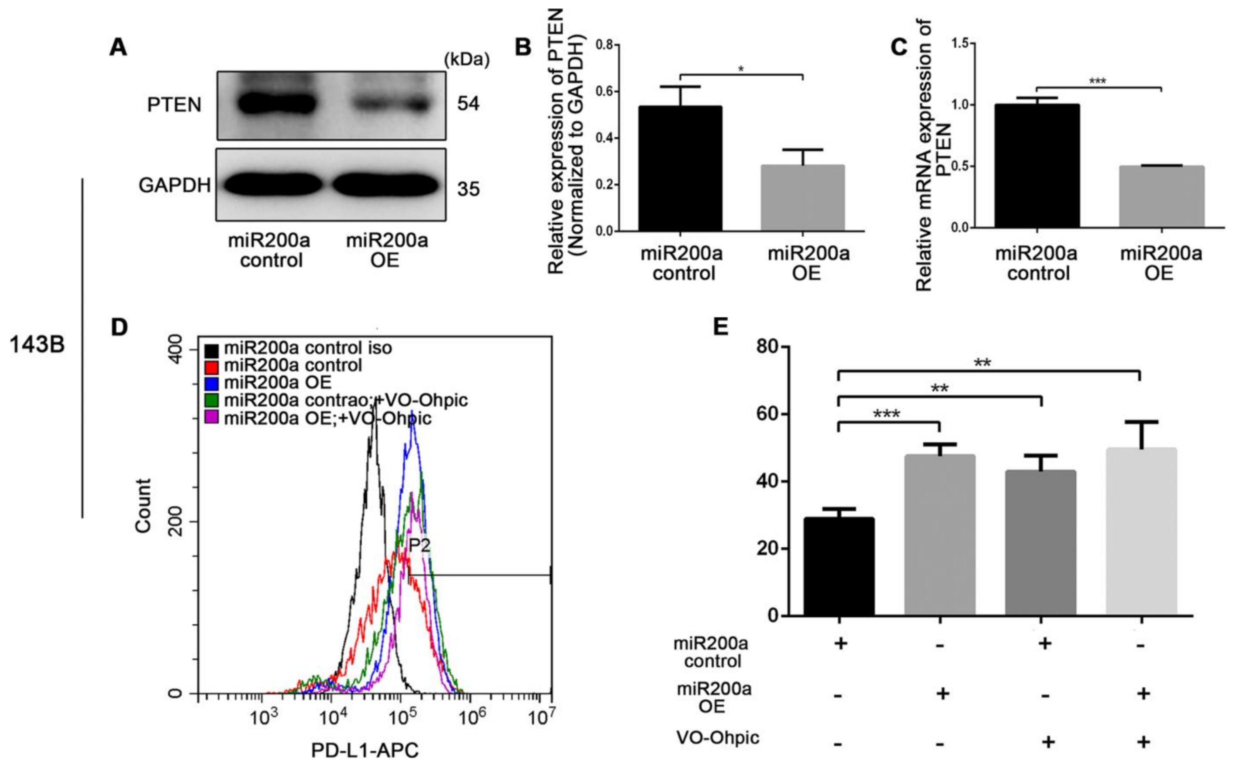

Supplementary Figure 5. miR-200a up-regulated PD-L1 expression by targeting PTEN in 143B. (**A**–**B**) Western blot analysis of PTEN in 143B miR-200a control and miR-200a OE. (**C**) qRT-PCR analysis of PTEN in 143B miR-200a control and miR-200a OE. (**D**–**E**) Flow cytometry analysis of PD-L1 expression of 143B miR-200a control and miR-200a OE after the addition of VO-Ohpic. *P<0.05, **P<0.01, ***P<0.001.

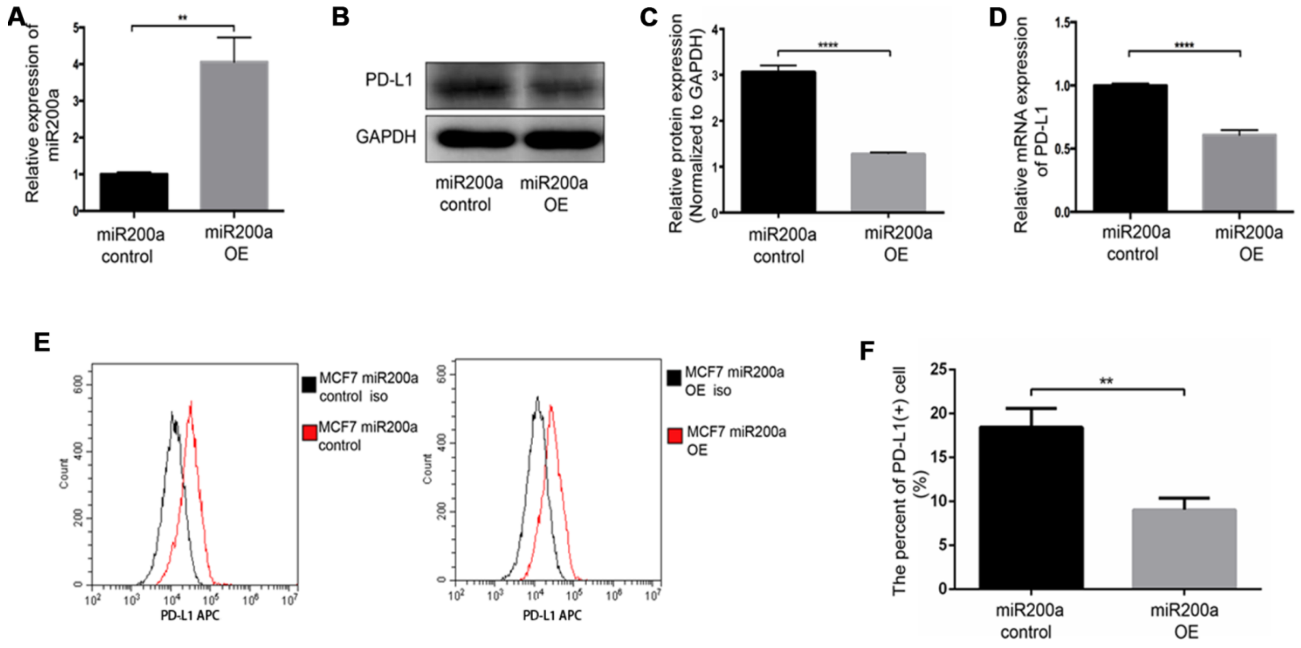

Supplementary Figure 6. miR-200a inhibited PD-L1 expression in MCF7. (**A**) qRT-PCR analysis of miR-200a in MCR7 miR-200a control and miR-200a OE. (**B**–**C**) Western blot analysis of PD-L1 in MCF7 miR-200a control and miR-200a OE. (**D**) qRT-PCR analysis of PD-L1 in MCF7 miR-200a control and miR-200a OE. (**E**–**F**) Flow cytometry analysis of PD-L1 in MCF7 miR-200a control and miR-200a OE. *P<0.05, **P<0.01, ***P<0.001.

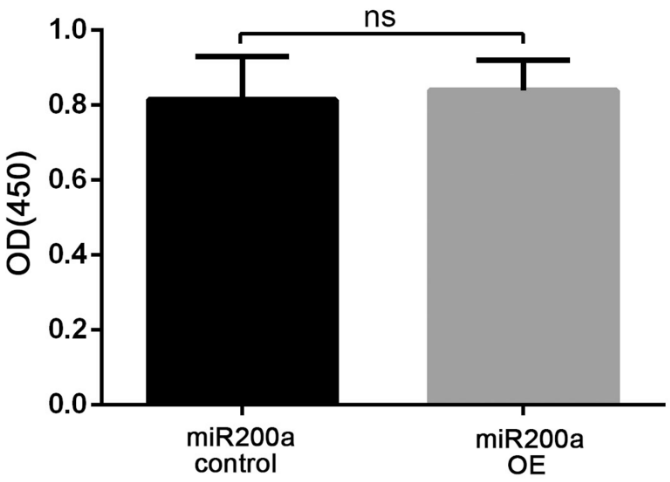

Supplementary Figure 7. Proliferative activity of K7 miR-200a control and miR-200a OE.
