# Supplementary material for: MicroRNA-200a induces immunosuppression by promoting PTEN-mediated PD-L1 upregulation in osteosarcoma
Source: Aging (Albany NY). 2020 Jan 24;12(2):1213–36. doi: 10.18632/aging.102679 (PMC7053609; doi:10.18632/aging.102679)
Supplement: Supplementary Table 1 [file aging-12-102679-s001..pdf]

SUPPLEMENTARY TABLE

Supplementary Table 1. 36 differentially expressed miRNAs from RNA-seq analyses of U2OSR2 and U2OS.

Gene	Fold change (U2OSR2-U2OS)	P values
hsa-miR-429	9.62048	0.000102
hsa-miR-1269a	-8.56904	0.001145
hsa-miR-3616-5p	-8.25226	0.00227
hsa-miR-891a-5p	6.217151	0.002303
hsa-miR-7854-3p	-5.33468	0.002592
hsa-miR-200a	6.704126	0.003595
hsa-miR-3689e	9.496191	0.007116
hsa-miR-3689a-5p	9.247936	0.008183
hsa-miR-3157-5p	7.851183	0.011445
hsa-miR-3149	-4.62893	0.012796
hsa-miR-642b-3p	-6.93741	0.015153
hsa-miR-7978	-4.2548	0.021055
hsa-miR-182-3p	5.820626	0.026538
hsa-miR-6843-3p	-2.79738	0.026727
hsa-miR-3157-3p	5.662572	0.026747
hsa-miR-183-3p	5.734097	0.027674
hsa-miR-3139	-7.12858	0.029658
hsa-miR-137-5p	-4.2046	0.030644
hsa-miR-4421	-7.76872	0.030777
hsa-miR-1252-5p	-6.71698	0.031086
hsa-miR-4707-5p	-3.05638	0.03227
hsa-miR-3660	2.849768	0.032505
hsa-miR-378e	3.183459	0.035972
hsa-miR-182-5p	5.474143	0.036141
hsa-miR-551a	6.305163	0.036888
hsa-miR-598-3p	-6.33812	0.038899
hsa-miR-4649-5p	-7.12858	0.039266
hsa-miR-3940-5p	-2.85583	0.040683
hsa-miR-183-5p	5.301127	0.045182
hsa-miR-6870-3p	-3.83141	0.045426
hsa-miR-1295a	7.222347	0.04657
hsa-miR-4695-3p	2.653211	0.047519
hsa-miR-3085-3p	-6.8314	0.048132
hsa-miR-6505-5p	2.74565	0.048341
hsa-miR-6889-3p	2.733657	0.049565
hsa-miR-483-3p	-6.34701	0.04996
